# Expression of nectin-4 in squamous cell carcinoma of prostate: a pilot study and case report

**DOI:** 10.3389/fonc.2026.1914312

**Published:** 2026-08-31

**Authors:** Xizhen Zhao, Fang Yu, Rui Zhou, Jiantao Xiao, Jianjun Fang, Tao Liu, Zhonghua Yang

**Affiliations:** 1 Department of Urology, Zhongnan Hospital of Wuhan University, Wuhan, China; 2 Department of Pathology, Zhongnan Hospital of Wuhan University, Wuhan, China; 3 Shanghai Jiao Tong University School of Medicine Affiliated Renji Hospital, Shanghai, China

**Keywords:** nectin 4, precision medicine, prostate cancer, squamous cell carcinoma of prostate, targeted therapy

## Abstract

**Background:**

Squamous cell carcinoma of the prostate (SCCP) is a rare subtype of prostate cancer with a poor prognosis, and no consensus exists regarding the treatment of SCCP. Nectin-4, a cell adhesion molecule predominantly expressed in a wide variety of cancers, emerged as a promising therapeutic target due to its over expression.

**Case presentation:**

A 69-year-old male patient received transurethral resection of the prostate for lower urinary tract symptoms (LUTS) and was diagnosed with prostatic acinar adenocarcinoma. Androgen deprivation therapy (ADT) was administered, and 22 months later, the patient presented with LUTS again, and radical prostatectomy (RP) was performed; subsequent pathologic examination revealed mixed prostatic carcinoma, with the squamous and glandular components accounting for 70% and 30%, respectively. Immunohistochemical (IHC) staining of the mixed carcinoma tissue from the RP specimen demonstrated strong immunoreactivity for Nectin-4 exclusively in the squamous component, not the glandular component. A retrospective assay of seven additional SCCP specimens from our center showed the same result. In the current case, although apalutamide, a novel androgen receptor inhibitor, in combination with ADT and pelvic radiotherapy was administered 1 month postoperatively, the patient experienced both biochemical and radiographic progression.

**Conclusion:**

This is the first report of a case of hormonal therapy-induced SCCP, in which Nectin-4 was expressed exclusively in the squamous component, not the glandular component. Nectin-4 might be a therapeutic target for SCCP.

## Introduction

Squamous cell carcinoma of the prostate (SCCP) is a rare subtype of prostate cancer with a poor prognosis, accounting for less than 1% of all prostate cancer cases (1). However, an increasing number of SCCP cases are being diagnosed as the use of novel androgen receptor pathway inhibitor (ARPI) therapy increases, which means that SCCP might be induced by hormonal therapy, especially ARPI therapy. Given the rarity of this disease, there is no consensus regarding the treatment. Personalized or precision medicine based on special targets might be a potential alternative. Nectin-4 is a cell adhesion molecule predominantly expressed in a wide variety of cancers (2). The success of an antibody-drug conjugate (ADC) targeting Nectin-4, namely enfortumab vedotin (EV), in the treatment of urothelial carcinoma (UC) makes the expression pattern of Nectin-4 across other malignancies an important consideration (3). In the current study, we report a case of treatment-induced SCCP and explore the expression of Nectin-4 in eight cases from our center.

## Case presentation

**Figure 1:**
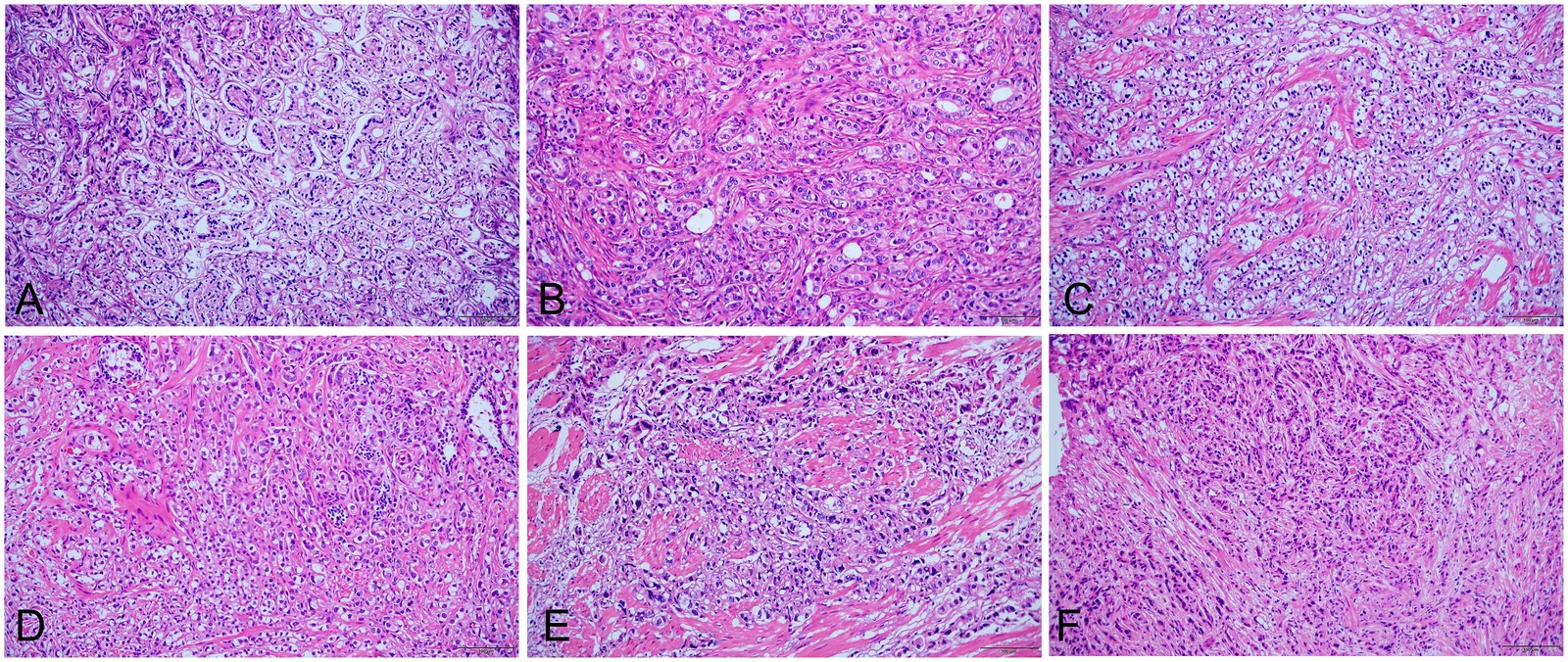
Prostatic acinar adenocarcinoma in TURP tissue. Gleason pattern 3 showed individual well-formed glands **(A)**. Gleason pattern 4 showed poorly formed glands **(B, C)** and some tumor cells with clear intracytoplasmic vacuoles **(C)**. Various morphologic subpatterns of Gleason pattern 5 were observed. A sheet of tumor cells with intracytoplasmic clear vacuoles exhibited signet ring cell-like morphology **(D)**. The tumor cells showed marked pleomorphism and infiltrative growth within the muscular layer **(E)**. Tumor cells with a significantly increased nuclear-to-cytoplasmic ratio were arranged in a cord-like pattern **(F)**.

A 69-year-old male patient underwent transurethral resection of the prostate (TURP) for lower urinary tract symptoms (LUTS) with a prostate-specific antigen (PSA) level of 9.61 ng/ml in September 2023. Postoperative pathologic examination revealed acinar adenocarcinoma [Gleason score 5 + 4 and the International Society of Urological Pathology (ISUP) grade group 5/5], with the tumor accounting for 90% of the submitted specimens (**Figure 1**). Additional examinations, including a bone scan and chest, abdominal, and pelvic CT scans, detected no distant metastasis. Then androgen deprivation therapy (ADT) was administered, and PSA decreased to an undetectable level but rose to 1.39 ng/mL and 2.45 ng/mL 18 and 19 months later, respectively. Then the patient was diagnosed with non-metastatic castration-resistant prostate cancer (nmCRPC), and abiraterone acetate plus prednisone (AAP) were administered. The PSA level decreased slowly and began to increase again only 3 months later, and the LUTS deteriorated.

## Results

**Figure 2:**
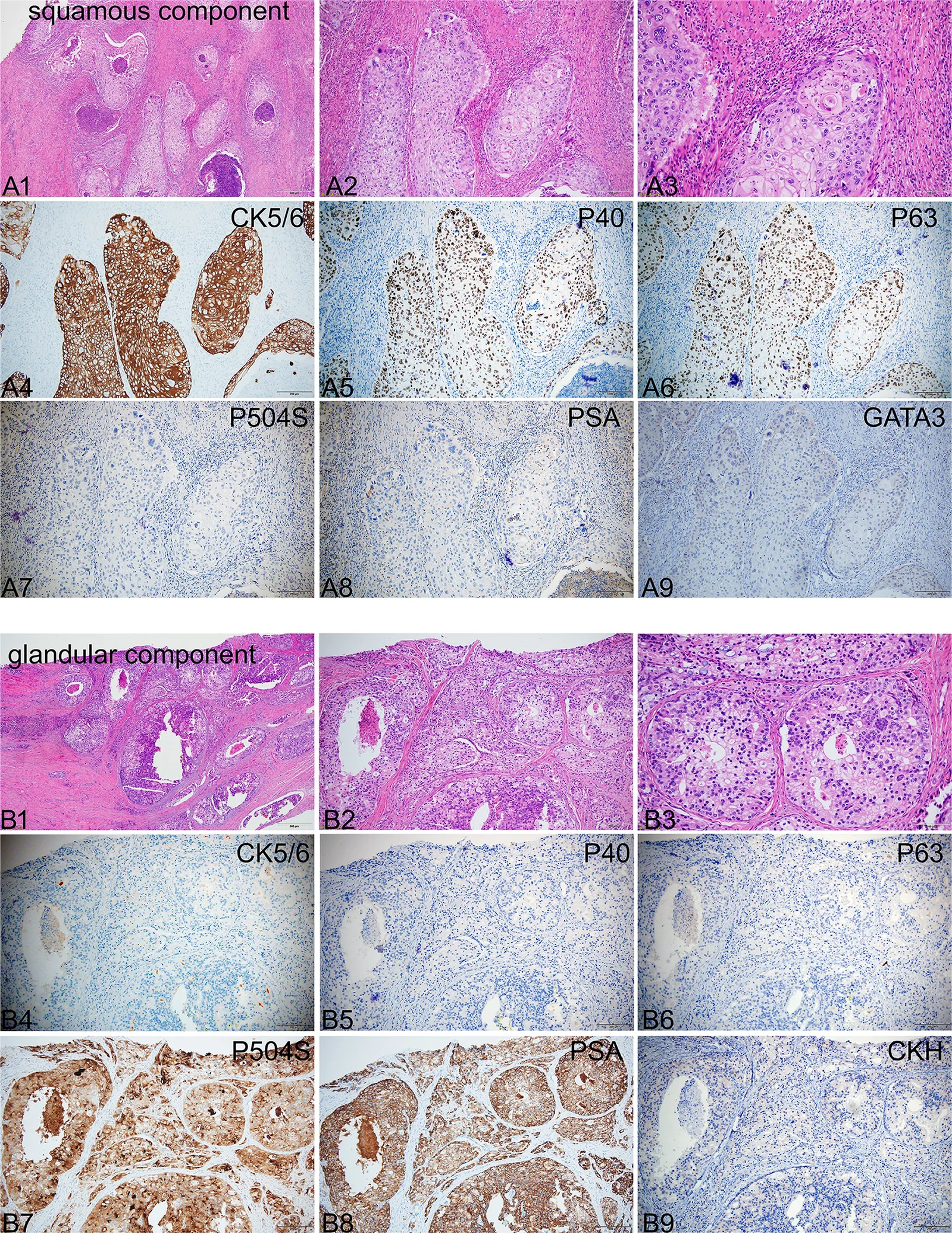
Adenosquamous carcinoma of the prostate. **(A)** The squamous component. The tumor cells in the squamous component were predominantly polygonal with clear cytoplasm (H&E staining, A1 ×40, A2 ×100, A3 ×200). Immunohistochemical stains (×100) confirmed the diagnosis of squamous cell carcinoma: CK5/6, P40, and P63 (A4–A6); the tumor cells were negative for PSA, P504S, and GATA3 (A7–A9). **(B)** The glandular component. The glandular component presented a cribriform structure with or without central necrosis (H&E staining, B1 ×40, B2 ×100, B3 ×200). The tumor cells in these areas were negative for CK5/6 (B4) and P40 (B5) but were positive for PSA (B7) and P504S (B8). The absence of expression of the basal cell markers P63 (B6) and CKH (B9) excluded intraductal carcinoma of the prostate.

**Figure 3:**
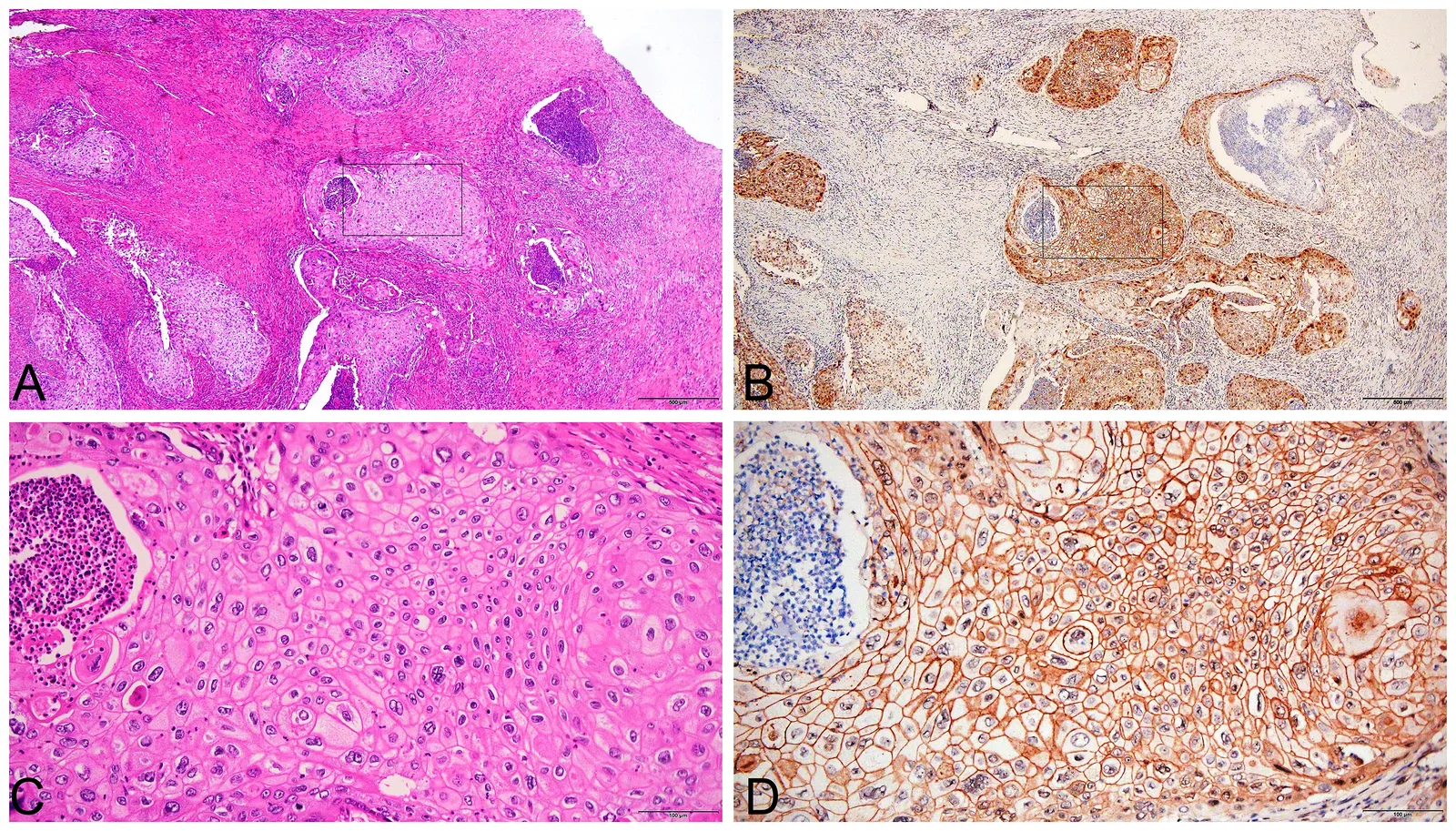
Membranous Nectin-4 expression patterns assessed using immunohistochemistry in a radical prostatectomy specimen. H&E staining **(A, C)** and membranous immunohistochemical staining for Nectin-4 **(B, D)** are shown for SCCP in the radical prostatectomy specimen (at magnifications of ×100 and ×500, respectively). Strong membranous Nectin-4 expression was detected in squamous cell carcinoma but not in the glandular component or the surrounding benign prostate gland **(B, D)**.

After distant metastasis was excluded using prostate-specific membrane antigen (PSMA) PET/CT, radical prostatectomy (RP) was performed, and the final pathologic examination revealed mixed prostatic carcinoma, in which SCCP and acinar adenocarcinoma accounted for 70% and 30% of the total tumor, respectively (Gleason score 5 + 5, ISUP 5/5, ypT3bN1Mx) (**Figure 2**). To explore the expression of Nectin-4 in SCCP, immunohistochemical (IHC) staining for Nectin-4 was performed. An anti-Nectin-4 monoclonal antibody (ab192033, Abcam, Cambridge, UK, at a dilution of 1:800, incubated for 40 min at 37 °C) was used as the primary antibody. Antigen recovery was performed using CC1 buffer from Ventana Medical Systems, Tucson, AZ, USA, for 64 min at 100 °C. IHC staining for P40, cytokeratin 5/6 (CK5/6), and androgen receptor (AR) was performed according to the protocol for each antibody. Given the small sample size, a manual membrane-specific H-score was determined by pathologists. At least 100 evaluable tumor cells were counted per high-power field, and only membranous staining intensity was scored as 0, 1+, 2+, or 3 +. H-score = 3 × (%3+) + 2 × (%2+) + 1 × (%1+), range 0–300. Cytoplasmic staining was excluded from evaluation. As shown in **Figure 3**, Nectin-4 can be seen exclusively in the SCCP component but not in the acinar adenocarcinoma component of the mixed tumor. In the SCCP component, about 90% of cancer cells expressed Nectin-4 on the membrane with a staining intensity of 3 +.

**Figure 4:**
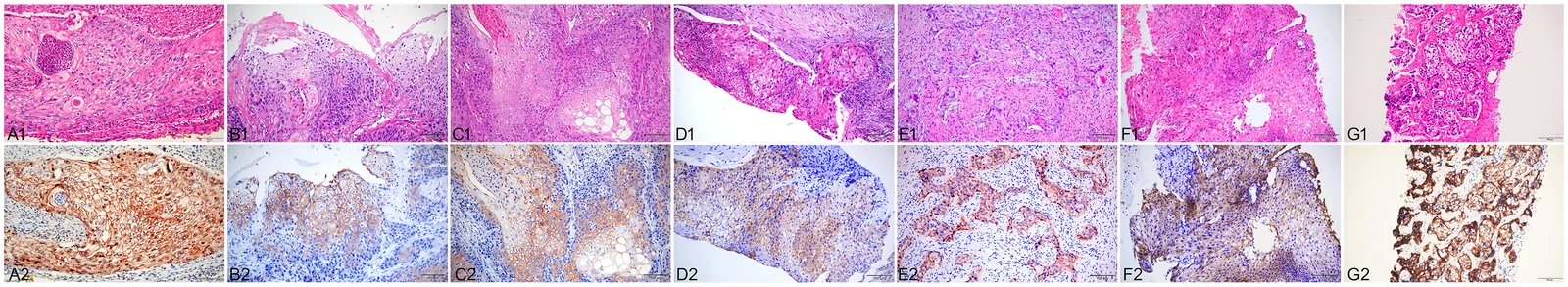
Expression of Nectin-4 in seven additional cases of SCCP. H&E staining (the top row) and IHC staining for Nectin-4 (the bottom row) are shown for additional cases of SCCP from our center. The majority of SCCP cells (60%–100%) in the mixed carcinomas expressed Nectin-4. Based on staining intensity and the proportion of positively stained cells, the H-scores of cases A–G were 260, 160, 170, 144, 90, 115, and 290, respectively.

The PSA level decreased from 3.89 ng/mL 8 hours before RP to 0.43 ng/mL 7 days postoperatively. The patient recovered urinary continence 1 month later, and pelvic radiotherapy at 66 Gy, in combination with ADT and apalutamide, an ARPI, was administered. Three months later, the PSA level rose to 80 ng/mL, and PSMA PET/CT and fluorodeoxyglucose (FDG) PET/CT scans revealed extensive lymph node metastasis, including supraclavicular, retroperitoneal, and external iliac lymph nodes (**Figure 4**).

Although Nectin-4 was overexpressed in SCCP and EV plus pembrolizumab led to significantly better event-free and overall survival outcomes in local/locally advanced (4) and advanced urothelial carcinoma (5), respectively, we did not prescribe EV in the current case for ethical reasons.

## Discussion

Although occasional cases of *de novo* SCCP have been reported, more than 50% of cases are reported to arise after treatment, such as androgen deprivation therapy (ADT) or radiotherapy (6). The current patient received hormonal therapy after TURP and was diagnosed with prostate cancer. However, after SCCP was diagnosed following RP, the TURP sample was reviewed by two experienced pathologists, and no evidence of SCCP was found. Furthermore, in the RP sample, the areas of adenocarcinoma and squamous cell carcinoma were distinctly separated, with the squamous and glandular components accounting for 70% and 30%, respectively. The tumor cells in the squamous component were predominantly polygonal with clear cytoplasm. The nuclei of these tumor cells displayed coarse chromatin and prominent nucleoli. Intranuclear pseudo inclusions were observed in a few tumor cells, and atypical mitoses were easily identifiable. Atypical concentric keratinization can be found in some areas (**Figure 2**, A1–A3). The squamous component showed diffuse immunoreactivity for CK5/6, P40, and P63 (**Figure 2**, A4–A6), whereas it was negative for the prostatic markers P504S and PSA and for the urothelial carcinoma marker GATA3 (**Figure 2**, B1–B3). The tumor cells in the glandular component were positive for P504S and PSA (**Figure 2**, B4–B5), while being negative for CK5/6, P40, P63, and the basal cell marker high molecular weight cytokeratin (CKH) (**Figure 2**, B6–B9). All of these demonstrated that this case of SCCP might have been induced by hormonal therapy. Whole-exome sequencing (WES) can help to verify the origin of both tumor components.

SCCP is more likely to have metastasized to bone, liver, or lungs at the time of diagnosis (7). As in the current case, radiography revealed only regional lymph node metastasis, without signs of bone or visceral metastasis when radical prostatectomy was performed. Furthermore, after second-line hormonal therapy in combination with postoperative radiotherapy, the patient developed extensive lymph node metastasis but no bone or visceral metastasis (data not shown).

Currently, there is no consensus regarding the treatment of SCCP because of the rarity of this disease. The latest and largest retrospective review of patients diagnosed with primary SCCP between 2000 and 2018 showed a 5-year overall survival (OS) of 24% and a median survival of 1.2 years, respectively (8). Furthermore, an analysis of 5-year OS stratified by treatment modality revealed no statistically significant difference among treatment modalities, including surgery, radiotherapy, and chemotherapy (8). So personalized or precision medicine based on a special target might be a potential alternative.

Nectin-4 emerged as a promising therapeutic target due to its overexpression in a wide variety of cancers, including bladder cancer. As for squamous cell carcinoma (SCC), recent studies by Stuebs FA et al. reported the expression of Nectin-4 in several kinds of SCCs, including vulvar squamous cell carcinoma (VSCC) (9), penile squamous cell carcinoma (10), oropharyngeal squamous cell carcinoma (OSCC) (11), and vaginal squamous cell carcinoma (12). However, investigations of the expression and role of Nectin-4 in prostate cancer showed discrepant findings (13, 14). And the antibody-drug conjugate (ADC) EV targeting Nectin-4 led to significant growth inhibition in the Nectin-4-expressing cell lines, including 22Rv1 and LNCaP, while Nectin-4-negative PC-3 cells were shown to be resistant to EV treatment. This means Nectin-4 should be a potential therapeutic target for prostate cancer in a biomarker-stratified subgroup (13).

Other studies have shown that Nectin-4 can be detected both on the membrane and in the cytoplasm (14). In the current case, Nectin-4 was exclusively expressed on the membrane of most SCCP cells (about 90%) with an intensity of 3+ (**Figure 3**), which was confirmed by a subsequent retrospective study on samples from seven additional SCCP cases from our center (**Figure 4**). Since the cell uptake of ADCs and other therapies likely requires binding to the membrane, it is plausible that Nectin-4 expression should be preferentially assessed for membranous expression rather than combined membranous/cytoplasmic expression in order to determine the benefit of therapy. On the other hand, a study in urothelial cancer linked lineage plasticity with Nectin-4 expression, and the squamous subtype retained a moderate Nectin-4 level that can be used as a therapeutic target for FDA-approved EV (15). We also explored the expression of Nectin-4 in a small number of samples from other prostate cancer subtypes, including neuroendocrine prostate cancer (NEPC) and ductal adenocarcinoma of the prostate (DAP), and detected no signs of expression of Nectin-4 in these subtypes (data not shown). This means Nectin-4 might be a therapeutic target for SCCP, but not for acinar adenocarcinoma, NEPC, or DAP.

This is the first report of expression of Nectin-4 in the SCCP components of mixed tumors from eight cases. With an increasing number of SCCP cases being diagnosed as the use of novel ARPI therapy increases, Nectin-4, a promising therapeutic target in bladder cancer, might be a potential therapeutic target in SCCP because of its overexpression and exclusive expression. Although most SCCP cells in the eight cases from our center expressed Nectin-4, given the small sample size and lack of *in vivo* or *in vitro* data, the findings should be interpreted with caution. Further studies are needed to verify the expression mode of Nectin-4 in larger samples and the anti-cancer effect of EV in SCCP.

## Data Availability

The raw data supporting the conclusions of this article will be made available by the authors, without undue reservation.
